# Supplementary material for: Feasibility characteristics of wrist-worn fitness trackers in health status monitoring for post-COVID patients in remote and rural areas
Source: PLOS Digit Health. 2024 Aug 22;3(8):e0000571. doi: 10.1371/journal.pdig.0000571 (PMC11340956; doi:10.1371/journal.pdig.0000571)
Supplement: S1 Table — (DOCX) [file pdig.0000571.s001.docx]

| **Symptom** | **Mean difference after 8 weeks** | **P-value** |
| --- | --- | --- |
| Constitutional | 0.18 | 0.63 |
| Neuro | 0.20 | 0.37 |
| Gastrointestinal | -0.03 | 0.17 |
| Respiratory | -0.23 | 0.03 |
| Cardiovascular | -0.39 | 0.09 |
| Mood | -2.81 | 0.66 |
| Anxiety | -2.96 | 0.86 |
| Stress | -4.36 | 0.45 |
| Total Steps | -108.4 | 0.18 |
| Resting Heart Rate (bpm) | -4.31 | 0.32 |
| Average Heart Rate (bpm) | -5.99 | 0.10 |
