# Supplementary material for: Feasibility characteristics of wrist-worn fitness trackers in health status monitoring for post-COVID patients in remote and rural areas
Source: PLOS Digit Health. 2024 Aug 22;3(8):e0000571. doi: 10.1371/journal.pdig.0000571 (PMC11340956; doi:10.1371/journal.pdig.0000571)
Supplement: S2 Table — (DOCX) [file pdig.0000571.s002.docx]

| **Complications for Hospitalized Patients** | **Frequency** |
| --- | --- |
| Cardiovascular (ex. acute MI, atrial fibrillation, pulmonary embolism) | 5 |
| Infectious (non-respiratory) (ex. urinary tract infection, sepsis) | 3 |
| Excretory (ex. acute kidney injury) | 2 |
| Metabolic (alkalosis, hypokalemia) | 2 |
| Hematologic (ex. bleeding) | 2 |
| **Medical comorbidities** | **Frequency** |
| Arrhythmia | 1 |
| Pulmonary Circulation Disease | 2 |
| Hypertension | 2 |
| Chronic Pulmonary Disease | 1 |
| Peptic Ulcer Disease | 1 |
| Fluid and Electrolyte Diseases | 1 |
| **Medications** | **Frequency** |
| **Number of Medications** | **4.3 [SD=3.1]** |
| Alimentary Tract | 3 |
| Blood and blood forming | 0 |
| Cardiovascular | 4 |
| Dermatologic | 0 |
| Genitourinary | 3 |
| Hormonal | 5 |
| Anti-Infective | 6 |
| Antineoplastic | 0 |
| Musculoskeletal | 2 |
| Nervous system | 8 |
| Antiparasitic | 0 |
| Respiratory | 5 |
| Sensory | 0 |
| Various (i.e. others) | 0 |
| **Physical Symptoms (Score 0-4, [SD])** | |
| ***Constitutional (6 symptoms)*** | 1.43[0.83] |
| Fatigue | 2.46 [1.18] |
| Aches and pains | 1.45 [1.32] |
| Muscle weakness | 1.32 [1.02] |
| Poor sleep | 1.72 [0.98] |
| Fever | 0.04 [0.09] |
| Feeling Generally Unwell | 1.60 [1.52] |
| ***Gastrointestinal (3 symptoms)*** | 0.31[0.55] |
| Nausea and Vomiting | 0.34 [0.62] |
| Diarrhea | 0.12 [0.28] |
| Abdominal Pain | 0.47 [0.88] |
| ***Neurological (3 symptoms)*** | 0.61[0.62] |
| Headache | 1.50 [1.37] |
| Loss of taste | 0.17 [0.47] |
| Loss of smell | 0.16 [0.51] |
| ***Respiratory (4 symptoms)*** | 0.90[0.55] |
| Cough | 0.92 [0.96] |
| Shortness of Breath | 1.70 [1.12] |
| Runny Nose | 0.54 [0.54] |
| Sore Throat | 0.47 [0.69] |
| ***Cardiovascular (2 symptoms)*** | 0.68[0.90] |
| Chest Pain | 0.83 [1.12] |
| Palpitations | 0.54 [0.75] |
| **Mental health Symptoms** | |
| General Anxiety Disorder-7 (out of 21) | 16.3 [7.0] |
| Patient Health Questionnaire-9 (out of 27) | 20.2 [8.2] |
| Perceived Stress Scale (out of 40) | 29.7 [5.3] |
| **Baseline technology and health literacy scores** | |
| Tech comfort (7-items, avg score [0-5]) | 3.59 |
| Health Literacy (4-items, avg score [0-5]) | 3.68 |
