# Supplementary material for: Feasibility characteristics of wrist-worn fitness trackers in health status monitoring for post-COVID patients in remote and rural areas
Source: PLOS Digit Health. 2024 Aug 22;3(8):e0000571. doi: 10.1371/journal.pdig.0000571 (PMC11340956; doi:10.1371/journal.pdig.0000571)
Supplement: S3 Table — (DOCX) [file pdig.0000571.s003.docx]

| **Factor** | **Correlation** | **P-value** |
| --- | --- | --- |
| Age | -0.27 | 0.46 |
| Admission Length | -0.38 | 0.28 |
| Symptoms Survey Response Rate | 0.67 | 0.03 |
| Mental Health Survey Response Rate | 0.53 | 0.11 |
| Tech Readiness Scores | 0.38 | 0.28 |
| All Symptoms | -0.43 | 0.21 |
| Generalized Anxiety Disorder-7 | -0.59 | 0.07 |
| Patient Health Questionnaire 9 | -0.52 | 0.13 |
| Perceived Stress Scale | -0.61 | 0.06 |
