# Supplementary material for: Feasibility characteristics of wrist-worn fitness trackers in health status monitoring for post-COVID patients in remote and rural areas
Source: PLOS Digit Health. 2024 Aug 22;3(8):e0000571. doi: 10.1371/journal.pdig.0000571 (PMC11340956; doi:10.1371/journal.pdig.0000571)
Supplement: Attachment — Submitted filename: PLOS rebuttal letter.pdf [file pdig.0000571.s006.pdf]

Madeleine Wiebe, BSc, MSc, 2025 MD Candidate
University of Alberta
116 Street & 85 Ave
Edmonton, Alberta
T6G 2R3
Email: mcwiebe@ualberta.ca

To the editors of PLOS Digital Health and reviewers of this manuscript,

Thank you for your extremely helpful revisions for this manuscript. We have considered all these suggestions and included them as follows, beginning with the issues brought by reviewer one:

In the introduction, we have better defined our hypothesis, from Line 105-110. In the methods section, we have added clarity to our description of the study cohort, including information on the minimum and maximum age restrictions (Line 129), if comorbidities (including substance use disorders) were considered during recruitment (Lines 133-134), and if participants required a smartphone to be recruited (Lines 132-133). We have also clarified if researchers and/or clinicians were involved in screening for recruitment (Lines 128-131) and clarified that the wearable devices used in this study are consumer-grade wearables, rather than medical devices (Line 144). We elaborated more on the integration of qualitative and quantitiave data usage on from Lines 186-189. For the statistical analysis, we included the type of program used to perform this (Line 208-209), and why an assessment of power was not performed (Lines 209-211).

Of reviewer one's comments on the results section, we clarified why three participants withdrew consent from the study (Lines 234-237). Reviewer one's comment on adherence data potentially affected by poor positioning of device is now addressed in the discussion section as a study limitation (starting on Line 524). Finally, reviewer one commented that since Canada was entering the fifth wave of COVID-19 during the recruitment period and the rural setting may have been less affected by infection rate, would this affect recruitment? We feel that the main limiting factor in this study is the recruitment capacity of the Early Supported Discharge (ESD) program, rather than infection rate, which is now addressed starting on Line 472.

For the descriptive analysis, reviewer one requested to specify exact weeks being analyzed (ex. Weeks 1-4, weeks 5-8, 8+) and for more detailed description of within-participant statistical. changes. We have now clarified the descriptive analysis starting on lines 272. We have moved the discission of within-participant analyses to the participant associations section starting on line 286. Here, we present the statistical significance for respiratory symptoms and expand on trends in the text but elect to leave the full table in the supplement given space constraints.

Starting on Line 301 (Table 3), significant values have been bolded for easier reading. Figure 2 (Line 323) has also been optimized for easier interpretation. Reviewer one also raised the point

that comparison of those hospitalized verses not might be beneficial to include for discussion. This was not a major theme identified in thematic analysis of our qualitative data and meaningful group comparisons were not possible given our small sample size. Thus, we have chosen not to highlight differences between those hospitalized vs. not in the manuscript.

For the discussion, reviewer one raised the question that technological literacy was part of our inclusion criteria and may contribute to a selection bias for a younger cohort with pre-existing comfortability for these devices. A brief discussion on the impact of providing more technological support and the potential effects on results has been added to this section, starting on Line 460. We have also added a note on potential applications for machine learning in the discussion (Lines 394-396).

Of reviewer two's comments, clarity is added to the frequency at which the biosensor parameters were collected are found in Lines 158-159, reported in the methods section. Reviewer two also requested enhanced clarity of Figure 2 (Line 323), which is completed.

From the Editor's comments, we have incorporated all the helpful discussion points suggested in our limitations section. We have elaborated on the potential influence of using wearable devices originally designed for healthy individuals on a sick cohort (starting on Line 522), that companies may use automatic updates or data collection abilities without the consumer knowing (Line 532-538), and how the differences in metric calculation and device characteristics make it difficult to compare different models or brands of devices (Lines 534-536).

We hope that these changes answer any outstanding questions or concerns. Thank you for your consideration,

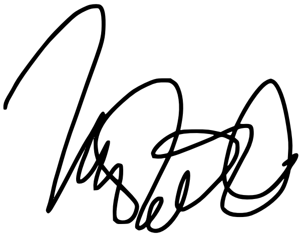

Madeleine Wiebe
